# Beneficial Fatty Acid Ratio of *Salvia hispanica* L. (Chia Seed) Potentially Inhibits Adipocyte Hypertrophy, and Decreases Adipokines Expression and Inflammation in Macrophage

**DOI:** 10.3390/foods9030368

**Published:** 2020-03-22

**Authors:** Subash-Babu Pandurangan, Salah A. Al-Maiman, Laila Naif Al-Harbi, Ali A. Alshatwi

**Affiliations:** Adipogenesis and Immunobiology Research Lab, Department of Food Science and Nutrition, College of Food Science and Agriculture, King Saud University, Riyadh 11451, Saudi Arabia; sbpandurangan@ksu.edu.sa (S.-B.P.); smaiman@ksu.edu.sa (S.A.A.-M.); lalharbi1@ksu.edu.sa (L.N.A.-H.)

**Keywords:** chia seed, ω-3 fatty acid, ω-6 fatty acid, inflammation, adipocyte, macrophage

## Abstract

The present study aimed to determine the role of *Salvia hispanica* L., (chia seed) fatty acid content in adipocyte lipid accumulation and human macrophage immunoregulatory potential. Chia seed fatty acid was extracted using hexane by the cold percolation method. A gas chromatography-mass spectrometry (GC-MS) analysis showed a 3:1 ratio of omega 3 and omega 6 fatty acid composition and it was more beneficial for human health. We treated it with increasing concentrations (0–6.4 μg/mL) of chia seed fatty acid extract to determine the cytotoxicity on the preadipocytes and macrophage; no significant cytotoxicity was observed. Chia seed, in 0.2 and 0.4 μg/mL doses, significantly arrested adipocyte hypertrophy and macrophage foam cell development. The gene expression levels of adipocyte confirmed the increased expression of adipocyte mitochondrial thermogenesis related genes, such as uncoupling protein-1 (UCP-1), peroxisome proliferator activated receptor gamma coactivator 1 alpha (PPARγC1α) and PR domain containing 16 (PRDM16); and the down regulated expression of the lipid synthesis related gene sterol regulatory element binding of protein-1c (SREBP-1c). In addition, adipogenesis related genes, such as the proliferator activated receptor γ (PPARγ) and CCAAT/enhancer binding protein (C/EBPα) expressions, have been down regulated by chia seed treatment. Macrophage treated with chia seed-treated adipocyte condition media significantly inhibited the obesity associated inflammatory genes and protein expression levels, such as monocyte chemo attractant protein-1 (MCP-1), prostaglandins E2, interleukin-6, plasminogen activator inhibitor-1 (PAI-1) and tumor necrosis factor-α (TNF-α). In conclusion, a 3:1 ratio of omega 3 and omega 6 fatty acid composition of chia seed fatty acid content potentially inhibits lipid accumulation, and enhanced fatty acid oxidation, via UCP-1 and PRDM16 expression. Macrophage recruitment to adipocyte and the development of obesity associated inflammation was suppressed by chia seeds.

## 1. Introduction

The omega 6 poly unsaturated fatty acids (PUFAs) associated with arachidonic acid might exhibit pro-inflammatory and pro-coagulatory functions. However, omega 3 fatty acid, such as decosahexaenoic acid and eicosapentaenoic acid, neutralizes the propagation of inflammation [1,2]. Current dietary patterns provide an abundant dietary source of omega-6 PUFAs and relatively poor abundance of omega-3 PUFAs [3]. Omega-6 fatty acids predominate over omega-3, which results in excessive circulating blood free fatty acids and hypertrophic white adipose tissues (WAT) [4]. It originates proinflammatory signals via leukotriene B4 (LTB4), the signal transducer and activator of transcription 6 (STAT6), interleukin-4 (IL-4) and tumor necrosis factor-α (TNF-α), which leads to an increased accumulation of immune cells in the WAT [5]. The imbalance among proinflammatory, proresolving fatty acids and eicosanoids appear to be the fundamental prominence for the progression of chronic inflammatory stage, insulin resistance and obesity associated inflammation [6]. Further excessive blood free fatty acids (FFAs) also stimulate the inflammation in macrophage linked with a risk of inflammatory cardiovascular disease [7,8,9].

Immune cells and adipocyte interaction have been well established in the progression of obesity and non-insulin dependent diabetes [10]. In obese and diabetic patients, peripheral blood mononuclear cells (monocytes and leucocytes) are identified with elevated inflammatory markers and a proinflammatory state [11]. In addition, the potential source of inflammatory molecules in adipose tissue might be due to the attraction, accumulation and activation of macrophage [12,13]. Activated macrophage further secrete a variety of cytokines and chemokine which regulate insulin resistance [14]. Adipose tissue activated macrophage secrete excessive of adipokine, including monocyte chemo attractant protein-1 (MCP-1) [9]. The released adipokine further recruit new macrophages in adipose tissue, and the infiltrated macrophage stabilize the inflammatory state via releasing numerous types of inflammatory mediators, such as cyclooxygenase-2 (COX-2), prostaglandins (PGE-2), interleukin-6, plasminogen activator inhibitor-1 (PAI-1) and tumor necrosis factor-α (TNF-α) [15].

Monocytes majorly recruited in hypertrophic adipocytes lead to the down regulation of anti-inflammatory cytokines and assist the progression of cardiovascular risk through decreasing the sensitivity to pharmacological antiaggregating agents [16]. Therefore, the potential way of inhibiting lipid accumulation or increased mitochondrial biogenesis in adipocyte leads to a significant reduction in the number of adipose tissue infiltrating macrophages in obese patients [17,18]. Therefore, dietary agents or anti-inflammatory compounds suppress the expression of proinflammatory factors in the adipose tissue, which may contribute to the improvement of obesity and its related disorders. Previous research confirmed that an increased quality diet (e.g., increased consumption of whole grains, fruit, vegetables, nuts/legumes, long-chain fats and PUFAs) favorably influences plasma biomarkers such as adiponectin [19].

*Salvia hispanica* L., (Chia) seeds have been well known as a rich source of omega 3 (α-Linoleic acid) fatty acid when compared to all the other plant sources [20]. Due to this fact, chia seeds have been used worldwide in diets for many years. In the present study, we aimed to determine the role of chia seed fatty acid content on lipid accumulation and mitochondrial thermogenesis in adipocyte. Further, chia seed-treated adipocyte condition media (secreted proteins) have been treated with macrophage to identify its polarization effect, which is linked with obesity associated inflammatory cytokine production. The present study majorly explores the interaction of chia seed fatty acid treated adipocyte microparticles on the regulation of macrophage immunomodulation.

## 2. Materials and Methods

### 2.1. Cell Lines and Chemicals

Human bone marrow-derived mesenchymal stem cells (hMSCs) have been purchased from American type culture collections (ATCC, USA). Dulbecco’s modified Eagle medium (DMEM), trypsin, EDTA and all cell culture materials have been purchased from Gibco, Paisley, UK. Additionally, 3-(4,5-dimethylthiazol-2-yl)-2,5-diphenyltetrazolium bromide (MTT), oil red O (ORO), nile red and histopaque were purchased from Sigma (St. Louis, MO, USA). Adipocyte differentiation factors such as insulin, rosiglitazone, dexamethasone (DEX), 3-isobutyl-1-methyl-xanthine (IBMX) and lipopolysaccharides (LPS) were bought from Sigma (St. Louis, MO, USA). Distilled water was obtained using the Milli-Q system (Millipore Laboratory, Bedford, MA, USA). The cytokine analyzing ELISA array kits were purchased from Qiagen (MEH004A, Qiagen, Hilden, Germany). The cDNA synthesis kit was purchased from Qiagen, Hilden, Germany. The SYBR Green PCR Master Mix was purchased from Qiagen, Hilden, Germany. Deionized water was obtained using a Direct-QUV 3 Millipore Water purification system (Millipore, Burlington, MA, USA). All other chemicals related to the molecular biology experiment were purchased from Sigma-Aldrich (St. Louis, MO, USA).

### 2.2. Chia Seed Collection and Fatty Acid Extraction

Chia seeds (*Salvia hispanica* L.) have been purchased from local hypermarkets. Chia seeds were ground using a commercial blender and immediately extracted with hexane using the cold percolation method. Briefly, 500 g of chia seed powder was soaked in 1.5 L of hexane and the content was vigorously shacked once in 3 h at room temperature for 72 h. Extracts were filtered through Whatman No. 1 filter paper and the solvent was evaporated using a rotary evaporator at 50 °C. The resulting viscous fatty oil concentrate was freeze-dried to ensure the complete removal of solvent and stored at −20 °C until needed for experiments.

### 2.3. Analysis of Chia Seed Fatty Acid Content

The phytochemical composition of chia seed hexane extract has been determined using the GCMS Agilent 7890 A, MS5975 system (Santa Clara, CA, USA) unit. It was equipped with a J&W-5MS fused silica capillary column containing 30 m × 0.25 mm, injected with Dose (ID) × 1 μm (% ID/g), with an injection volume of 3 μL (split ratio of 10:1), and coupled with helium as a carrier gas and detector with a mass selective; 250 °C and 280 °C will be the injector temperatures. The oven temperature was initially at 50 °C, held for 4 min and increased to 250 °C at a rate of 7 °C/min. LabSolution software (Shimadsu, Tokyo, Japan) will be used to control the operation of GCMS [8]. The mass spectra was obtained using the National Institute of Standard Technology (NIST-11) library.

### 2.4. Collection of Blood, Isolation of Monocyte and Stimulation of Monocyte to Macrophage Using Lipopolysaccharide (LPS)

Human peripheral blood monocytes (PBMC) were isolated from blood buffy coat provided by a healthy volunteer in King Saud University, Riyadh. The protocol and methodology have been sanctioned by the Institutional Review Board, institutional ethical committee, and the approval number is ‘E-19-4383‘. Briefly, 7 mL of whole blood was collected and immediately and gently transferred to EDTA added tubes, where it was carefully overlaid to a 3.5 mL of Histopaque-1077 (Sigma, St. Louis, MO, USA) containing centrifuge falcon tube and allowed to separate using centrifugation at 2000× *rpm* for 30 min. The mononuclear cells were carefully separated from the visible interface. The isolated adherent monocytes were washed with PBS, then seeded on plated culture containing 10% FBS, which contained DMEM medium (AG-Biochrom, Berlin, Germany). Monocytes were treated with 10 ng/mL of bacterial LPS for 24 h, to attain polarized macrophage. The stimulated polarized macrophage containing culture flask were kept inside the incubator at 37 °C with 5% CO_2_ and utilized for the experiment immediately.

### 2.5. Cell Culture and Adipocyte Differentiation

Early passaged human mesenchymal stem cells (hMSCs) were cultured (10^4^ cells/well) in Dulbecco’s modified Eagle’s medium (DMEM), supplemented with 10% fetal bovine serum (Gibco-Invitrogen, Gaithersburg, MD, USA) at 37 °C in 5% CO_2_ incubator. After 70% confluence of hMSCs, adipocyte differentiation was induced using a standard differentiation medium containing 1 μmol/L dexamethasone (DEX), 0.5 μmol/L 3-isobutyl-1-methyl-xanthine (IBMX) and 167 nmol/L human insulin containing 10% FBS/DMEM. After 72 h of adipocyte differentiation induction, the medium was replaced with maintenance medium containing 167 nmol/L insulin containing 10% FBS/DMEM for another 48 h. The differentiated preadipocytes were immediately utilized for experiments [21].

### 2.6. Cytotoxicity Assay

Increasing concentrations of chia seed fatty acid extract (0, 0.1, 0.2, 0.4, 0.8, 1.6, 3.2 and 6.4 μg/mL, dissolved in DMSO) have been treated and incubated for 24 h and 48 h with preadipocytes. The same increasing concentrations of chia seed extract have been treated with macrophages for 12 h and 24 h, respectively. At the end of the incubation, 3-(4,5-dimethylthiazol-2-yl)-2,5-diphenyltetrazolium bromide (MTT) was added to each well at a final concentration of 1 mg/mL and incubated for 4 h in the dark. The resulting formazan crystal was dissolved in 10% DMSO [22]. The plates with purple-colored formazan were absorbed (at λ = 570 nm) using a multiwell plate reader.

### 2.7. Experimental Design

To examine the effects of chia seed fatty acid extract on adipocyte’s fatty acid accumulation and maturation, chia seed was treated to human mesenchymal stem cells (hMSCs), along with adipocyte differentiation media, and maintained for 14 days. Briefly, on day 0, vehicle control, 0.1 μg/mL, 0.2 μg/mL and 0.4 μg/mL doses of chia seed fatty acid extract were treated to adipocyte differentiation induced hMSCs. On day 3, vehicle treated hMSCs were differentiated into preadipocytes and the differentiation medium was replaced with maintenance medium. On day 3, chia seed fatty acid extract treated preadipocytes were replaced with maintenance medium and treated again with the same doses of chia seed fatty acid extract, then maintained until day 6. From day 7, the media was replaced with maintenance medium once in 3 days until day 14. On day 14, the cell’s condition media (containing chia seed-treated adipocyte secreted proteins) were collected and the adherent cells were processed for lipid accumulation and gene expression analysis accordingly.

LPS stimulated macrophage cells were replaced with a 1:1 ratio of chia seed-treated adipocyte condition media and normal growth media and maintained for 12 h. In vehicle control group, both differentiated adipocyte and macrophages were treated with DMSO alone for the respective treatment period. At the end of the experiment, the supernatant was collected for the quantification of inflammatory cytokine and the cells were processed to synthesis total RNA, complementary DNA and processed for gene expression analysis.

### 2.8. Oil red O and Nile Red Staining Analysis

The level of lipid accumulation was determined using the modified method of Kim et al. [23]. At the end of experiment, vehicle control, 0.1, 0.2 and 0.4 μg/mL doses of chia seed-treated maturing adipocytes in 24-well culture plates were fixed with 4% formaldehyde, incubated with 200 μL of oil red O stain (stock: 500 mg of ORO in 100 mL of 100% isopropanol, and a working solution was prepared in 3:2 ratio of stock and 60% isopropanol in deionized water) for 60 min at room temperature. After incubation, the unbound oil red O was removed by repeated washing with PBS. Immediately, the images were analyzed using an inverted light and fluorescent microscope. After the image analysis, the stained cells were allowed to dry overnight and the oil stains were dissolved with isopropanol to measure the absorbance at 520 nm.

For the Nile red staining assay, the stock containing 5 mg of nile red dissolved in 1 mL of 100% acetone was used. The formaldehyde fixed vehicle control, 0.1, 0.2, 0.4 μg/mL chia seed fatty acid treated adipocytes were stained with 200 μL of fluorescence Nile red working solution (6 μL of stock Nile red dissolved in 1 mL of 40% isopropanol) for 30 min at room temperature. Then, the stained cells were analyzed using an inverted fluorescence microscope, and photographs were taken immediately.

### 2.9. Analysis of Triglyceride, Free Glycerol, High Density Lipoprotein, Low Density Lipoprotein and Lactate Dehydrogenase Activity

The quantity of triglyceride (TG), high density lipoprotein (HDL), low density lipoprotein (LDL) and free glycerol were measured in vehicle control, 0.2 μg/mL, 0.4 μg/mL of chia seed fatty acid and 6 Μm of orlistat treated adipocytes using the commercial kit method (Abcam, Austria) [24]. The lactate dehydrogenase (LDH) activity was determined using an enzymatic assay kit (Abcam, Austria). The protein content of chia seed-treated adipocyte cell was determined according to the Bradford method [25].

### 2.10. Mitochondrial Membrane Potential (JC-1 Staining) Asssay

To determine the mitochondrial efficiency on fatty acid metabolism, the JC-1 dye based mitochondrial membrane potential was analyzed. Vehicle control, 0.1, 0.2 and 0.4 μg/mL doses of chia seed fatty acid extract treated adipocytes in 24-well culture plates were selected for the JC-1 assay (Sigma, St. Louis, MO, USA). Briefly, equal volumes of culture medium and JC-1 staining solution were mixed and added to each well. After 20 min of incubation in the dark at 37 °C, cells were gently washed twice with 200 μL of JC-1 staining wash buffer at 4 °C. Fluorescence was observed using a fluorescence microscope (Biorad, Hercules, CA, USA) and images were captured.

### 2.11. Analysis of Gene Expression

Total RNA and cDNA have been synthesized using the Fastlane^®^ Cell cDNA kit (Qiagen, Hilden, Germany), using RT-PCR (Applied Biosystems, Foster City, CA, USA). Adipogenesis [CCAAT/enhancer binding protein-α(C/EBPα), peroxisome proliferator activated receptor γ (PPARγ), hormone sensitive lipase (HSL) and lipoprotein lipase (LPL)] and adipocyte mitochondrial thermogenesis related genes [Adiponectin, uncoupling protein- 1 (UCP-1), peroxisome proliferator activated receptor gamma coactivator 1 alpha (PPARγC1α), sterol regulatory element binding of protein-1c (SREBP1c) and PR domain containing 16 (PRDM16)], macrophage metabolic inflammation related genes [IL1β, IL12β1, IL-6, IL-4, IL-33, inhibitor of nuclear factor kappa B kinase subunit γ1 (IKBKγ1), nuclear factor kappa B (NF-κB), tumor necrosis factor-alpha (TNF-α), toll like receptor-4 (TLR-4) and transforming growth factor beta receptor (TGFBR2)] and the reference gene, β-actin, have been analyzed according to the method of Yuan et al. [26]. Primer sequences for the mentioned genes have been provided in the Table 1. We calculated ΔCt by the difference between Ct (treated) and Ct (control). The expression of mitochondrial oxidation and metabolic inflammation related genes were plotted using the expression of 2^−ΔΔCt^.

### 2.12. Quantification of Proteins by ELISA Method

Adipocyte secreted proteins, such as via leukotriene B4 (LTB4), LTB4, signal transducer and activator of transcription 6 (STAT6), interleukin-4 (IL-4) and tumor necrosis factor-α (TNF-α in adipocyte condition media and macrophage secreted proteins linked with metabolic inflammation related factors such as plasminogen activator inhibitor-1 (PAI-1), monocyte chemo attractant protein (MCP-1), prostaglandin E2 (PGE2), nuclear factor kappa B (NF-ĸB) and toll-like receptor-4 (TLR-4) have been assayed using highly sensitivity ELISA kits (Qiagen, Hilden, Germany). Results for the assayed proteins are normalized as a function of the total protein content and are expressed as pg/mg protein.

### 2.13. Statistical Analysis

Means, standard deviations and differences between treatments have been analyzed by a one-way analysis of variance (ANOVA), followed by Tukey’s test, using the SPSS version 11.5 software package (IBM, New York, USA) [27]. All the results were expressed as the mean ± SD and there were six replicates in each group (*n* = 6). For all comparisons, differences were considered statistically significant at *p* ≤ 0.01 and *p* ≤ 0.001.

## 3. Results and Discussion

**Figure 1:**
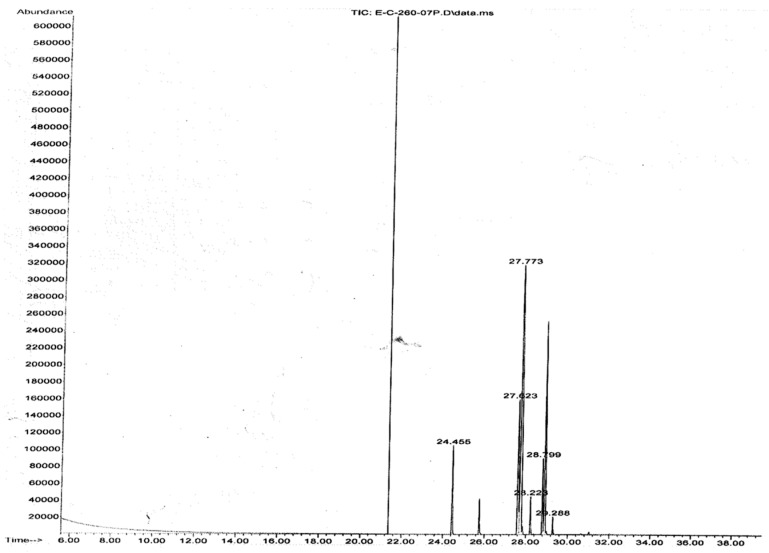
Gas chromatography-mass spectrometry (GC-MS) data for the fatty acid composition analysis of chia seed extract.

A GC-MS analysis of chia seed fatty acid content confirmed that 77.51% of the total component was omega 3 and omega 6 fatty acid (Figure 1). Overall, 56.16% of omega 3 fatty acid and 21.35% of omega 6 fatty acid have been determined in chia seed (Table 2). The GC-MS raw data, instrument method and conditions have been presented in the Supplementary Materials S1 and S2. In the present study, we confirmed that the percentage area and availability of omega 3 and omega 6 have been identified as a 3: 1 ratio in chia seed. The presence of the 3:1 ratio of omega 3 and omega 6 fatty acid was more beneficial for the control of immunoregulation in circulatory immune cells [7]. In our previous study, we confirmed the different ratio of omega 3 and omega 6 availability in edible oils directly associated with the immunomodulation in monocyte [8].

**Figure 2:**
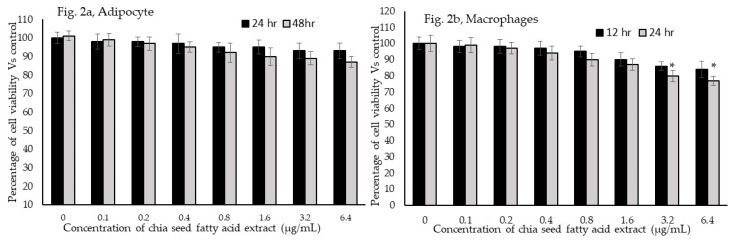
In vitro cytotoxic effect of chia seed extract on (**a**) human adipocyte (24 h and 48 h) and (**b**) macrophages (12 h and 24 h). Data are presented as the mean ± standard deviation (SD) (*n* = 6 in all the groups). * *p* < 0.05 vs. control.

In a cytotoxicity analysis, tested concentrations of chia seed extract showed much less cytotoxicity, both in preadipocytes and macrophage. In preadipocytes, 6.4 µg/mL of chia seed showed 7% and 13% reductions in the cell viability after 24 h and 48 h, respectively, and this is shown in Figure 2a. However, a higher concentration of 6.4 µg was tested, showing moderate toxicity in the macrophage, such as 16% in 12 h and 23% in 24 h (Figure 2b). According to our present observation, tested concentrations of chia seed did not produce IC_50_ or significant toxicity against preadipocytes or macrophages.

**Figure 3:**
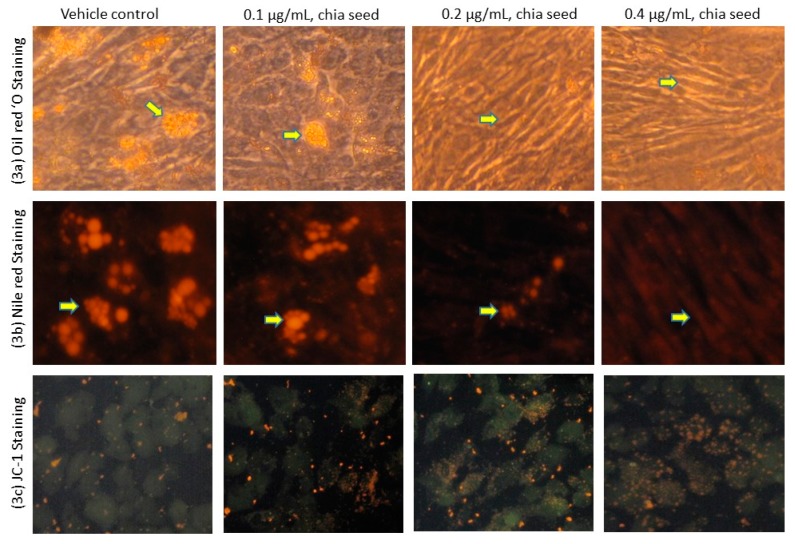
Results of oil red O (**a**), Nile red (**b**) and mitochondrial membrane potential, JC-1 (**c**) staining images (200×) of vehicle control and increasing doses of chia seed fatty acid extract treated adipocytes after 14 days.

In the present study, 0.1, 0.2 and 0.4 μg/mL concentrations of chia seed extract were selected for further study. Chia seed extract at a dose of 0.4 μg/mL significantly controlled adipocyte lipid accumulation was confirmed by oil red O (Figure 3a) and Nile red staining (Figure 3b). Moreover, a 0.4 µg/mL dose of chia seed-treated maturing adipocytes demonstrated restricted adipocyte hyperplasia, lipid accumulation, hypertrophic adipocytes and more spindle shaped adipocyte when compared to 0.1 µg/mL and 0.2 µg/mL doses of chia seed. The lower dose of chia seed inhibited lipid accumulation effectively when compared to the vehicle control.

JC-1 staining results also supported the present finding, such as the fact that lipophilic fluorophore forms J-aggregates, which are proportional to the mitochondrial membrane potential (MMP) (Figure 3c). JC-1 fluorescence images of each treatment group showing merged images of the red and green signals of the dye, corresponding to JC-1 in J-aggregates vs. monomeric form. We found that 0.4 μg/mL of chia seed showed high MMP, directly representing active mitochondrial thermogenesis. In this context, epigallocatechin [28,29] and aloe-emodin [30] have been reported for their anti-obesity potential via the inhibition of adipogenesis and the enhancement of fatty acid oxidation.

**Figure 4:**
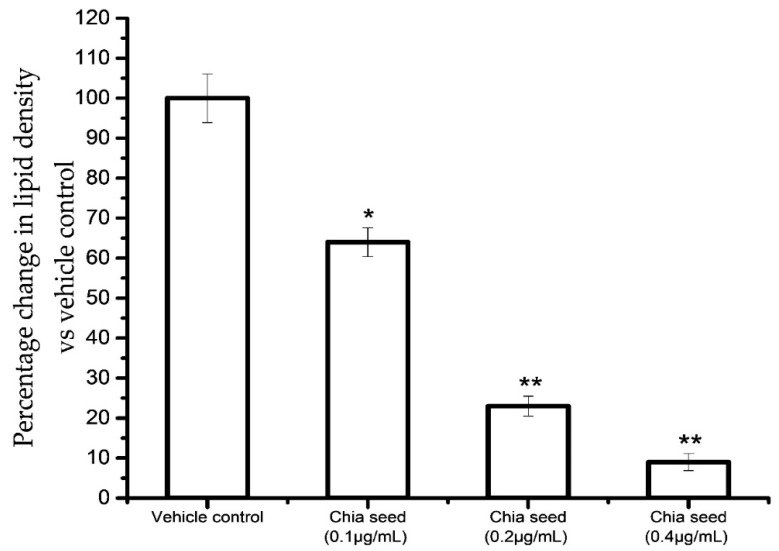
Relative density of oil red O extracted from chia seed fatty acid treated maturing adipocytes after 14 days. Data are expressed as the mean ± S.E.M. (*n* = 6). * *p* < 0.05 and ** *p* < 0.001 vs. vehicle control.

Figure 4 shows the results for the accumulated oil red O stain concentration in control and chia seed-treated maturing adipocytes after 14 days. The results confirmed that the chia seed fatty acid extract treatment significantly decreased oil red O stain concentration in a dose-dependent manner, such as 91% in 0.4 μg/mL (*p* ≤ 0.001), 77% in 0.2 μg/mL (*p* ≤ 0.001) and 46% in 0.1 μg/mL (*p* ≤ 0.05) of chis seed fatty acid when compared to vehicle control.

In addition, we found that triglyceride, free glycerol and LDL levels also decreased in chia seed-treated maturing adipocytes when compared to the vehicle control (Table 3). In this context, edible organic seed fatty oils and natural agents had a beneficial effect on the regulation of lipid accumulation and innate and acquired immunity in monocyte [31]. Hypertrophic adipocyte and immune cell interaction have been well proven for the progression of insulin resistance and non-insulin dependent diabetes [32]. The appropriate ratio of omega-6/omega-3 fatty acid is an important health determinant and decreases the inflammatory condition and cardiovascular disease [33,34]. Our present observation confirmed that the chia seed fatty acid ratio suppresses adipocyte lipogenesis and hypertrophy, which favor the suppression of proinflammatory cytokine formation in the adipose tissue.

In oil red O and Nile red staining, the vehicle control showing hypertrophic and high red fluorescence was directly propositional to the stored triglycerides. However, in 0.4 µg/mL of chia seed-treated cells showing controlled adipocyte maturation, there were less lipid accumulation and spindle-shaped adipocyte when compared to 0.1 µg/mL, 0.2 µg/mL of chia seed-treated and vehicle control cells. In JC-1 staining, vehicle control showing less J-aggregates proportional to less active mitochondria and mitochondrial potential (higher green monomer and lesser red monomers). However, a 0.4 µg/mL dose of chia seed treatment showing high J-aggregates (higher red monomer and less green monomer) is directly proportional to high active mitochondria (membrane potential) compared to 0.1 µg/mL and 0.2 µg/mL of chia seed-treated adipocytes.

**Figure 5:**
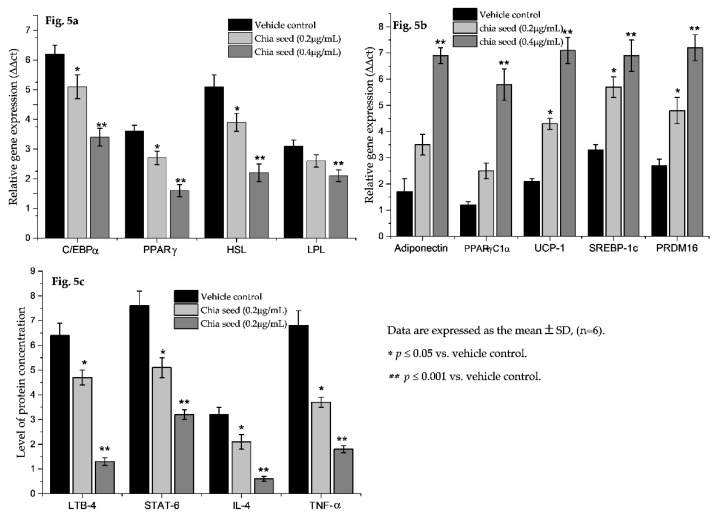
Effect of chia seed fatty acid extract on adipogenesis (**a**), adipocyte mitochondrial thermogenesis (**b**) related mRNA expression levels and protein levels (**c**) in adipocytes.

In the present study, chia seed fatty acid significantly decreased adipocyte hyperplasia related genes, such asC/EBPα, PPARγ, LPL and HSL, expression levels when compared to vehicle control (Figure 5a). Most notably, chia seed treatment significantly increased the expressions levels of adipocyte mitochondrial efficiency related genes such as adiponectin, UCP-1, PPARγC1α, SREBP1c and PRDM16 and these have been presented in Figure 5b. The increased gene expression levels for adipocyte mitochondrial β oxidation related factors, such as adiponectin, UCP-1, PPARγC1α, SREBP1c and PRDM16, have confirmed that chia seed fatty acid composition regulates lipolysis via mitochondria dependent fatty acid β oxidation. In addition, in vehicle control the down regulated expression levels of adipocyte hyperplasic and hypertrophic genes levels were not observed. However, chia seed fatty acid extract treatment potentially inhibits adipocyte maturation and lipid accumulation, which could be due to the availability of the 3:1 ratio of omega 3 and omega 6 fatty acid. The clinical significance of this observation reveals that the chia seed fatty acid extract significantly inhibited the adipocyte hyperplasia via arresting the C/EBPα and PPARγ mRNA expressions. Additionally, mitochondrial thermogenesis was increased in maturing adipocyte via the activation of adiponectin, PPARγC_1_α, PRDM16 and UCP-1 expressions. In this context, Ferreira et al. [35] have reported that the dietary intake of chia seed reverses a sucrose-rich diet-induced adipose tissue dysfunction and insulin resistance in rats. In addition, Chani et al. [36] reported that the long term intake of chia seed lowers the lipid deposit in hepatocytes and increases the intestinal muscle layer and crypt size in Sprague–Dawley rats.

A protein expression analysis of chia seed-treated adipocyte secreted microparticles showed decreased levels of insulin resistance and macrophage lipid accumulation related proteins such as LTB4, STAT6, IL-4 and TNF-α (Figure 5c). Most interestingly, insulin resistance- and obesity-associated immune disorder progressive proteins, such as, LTB-4R, STAT6, IL-4 and TNF-α levels, have been suppressed by chia seed treatment. Our findings are in line with Kang et al. [37] that punicalagin has been proven for its anti-obesity effect and obesity-induced inflammatory responses via the Nrf2/Keap1 Signaling Pathway.

**Figure 6:**
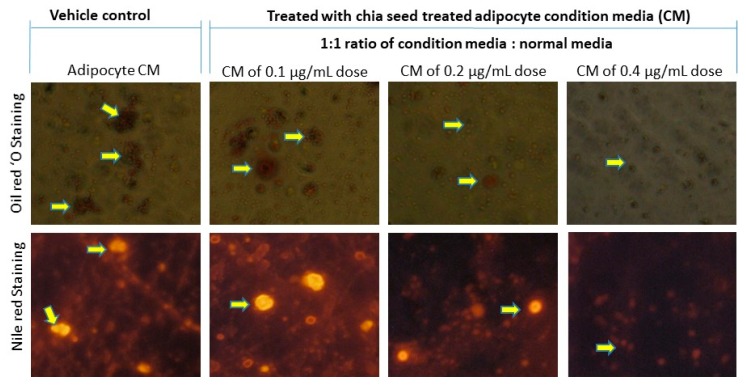
Oil red O and Nile red staining images of different doses of chia seed-treated adipocyte condition media-treated macrophages after 12 h.

**Figure 7:**
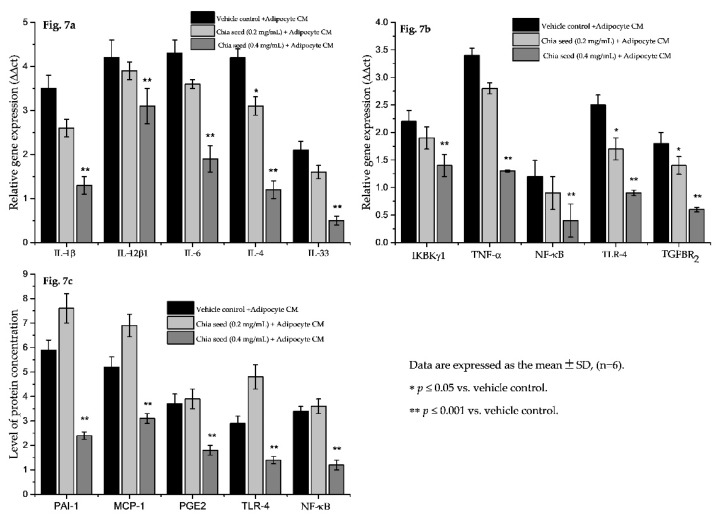
Changes in macrophage inflammatory marker related mRNA expression (**a**,**b**) and protein levels (**c**) of chia seed-treated adipocyte condition media-treated macrophage after 12 h.

Hypertrophic adipocyte secreted adipokines interact with macrophage and develop foam cells linked with atherosclerosis [38]. In the present study, chia seed-treated adipocyte condition media-treated macrophage were analyzed for foam cell formation using a florescence microscopic analysis. The florescent lipid staining of macrophage is shown in Figure 6, where we found increased foam cells or inflated macrophage in vehicle control adipocyte condition media-treated macrophage. However, 0.2 or 0.4 μg/mL doses of chia seed-treated adipocyte condition media effectively reduced the inflated macrophage and foam cell percentage. The ratio of proinflammatory and pro-resolving eicosanoids was desired to be the fundamental for the inhibition and control of the macrophage inflammatory response [2,6]. Furthermore, a gene expression analysis of chia seed-treated macrophage showed that the metabolic inflammation related genes IL1β, IL12β1, IL-6, IL-4, IL-33, IKBKγ1, NF-κB, TNFα, TLR-4 and TGFBR2 have been down regulated when compared to vehicle control (Figure 7a,b).

In addition, metabolic inflammation related proteins such as PAI-1, MCP-1, PGE2, TLR-4 and NF-ĸB were decreased after the use of chia seed fatty acid-treated adipocyte condition media-treated macrophages, when compared with vehicle control (Figure 7c). In vehicle control, we found an increase in the levels of the PAI-1, MCP-1, PGE2 and NF-ĸB inflammatory markers. They are mainly linked with the development and progression of foam cell formation and atherosclerotic lesion, which further contribute to atherosclerotic plaque instability and thrombus formation [38]. However, chia seed-treated adipocyte condition media-treated macrophage did not show a higher expression of macrophage colony stimulation factor or PGE2 level. In addition, the gene expression levels of IKBKγ1, TNFα, NF-κB, TNFα, TLR-4 and TGFBR2 also effectively suppressed in chia seed stimulated adipocyte condition media-treated macrophage.

The oil red O and Nile red image of macrophage of adipocyte condition media alone treated macrophage shows inflated, clumped and macrophage foam cells. The same features have been observed, even in 0.1 and 0.2 µg/mL of chia seed-treated condition media. However, 0.4 µg/mL of chia seed-treated adipocyte condition media inhibits inflated and macrophage foam cells formation by 100%.

The observed anti-obesity and immunoregulatory mechanistic effect of chia seed might be due to the availability of a 3:1 ratio of omega 3 and omega 6 fatty acid; it was more beneficial to human health. Schwarzkop et al. [39] have reported that the plasma levels of omega 3 and omega 6 are the precursor for inflammatory mediators in humans. The dietary availability of omega 3 fatty acid (α-linolenic acid) effectively arrests adipocyte hypertrophy and the origin of metabolic inflammation in adipocyte [35]. In addition, Rui et al. [40] have reported that chia seed decreased the senescence markers in adipose tissue by increasing the phosphorylated AMPK levels. The diminished phosphorylation of AMPK, linked with reduced mitochondrial β oxidation, increased triglyceride storage and oxidative stress end with insulin resistance and inflammation [41]. In the present study, the observed lipid lowering effect of chia seed might be linked with the increased expression of PPARγC1α, PRDM16, UCP-1 and adiponectin level, that stimulate mitochondrial β oxidation and deplete triglyceride storage. Furthermore, a metabolically active adipocyte inhibits the origin of adipokine and proinflammatory cytokines associated with the macrophage colony stimulating factor and atherosclerotic progression.

## 4. Conclusions

In conclusion, chia seed has a health beneficial 3:1 ratio of omega 3 and omega 6 fatty acid content. Chia seed fatty acid treated to maturing adipocyte effectively decreases lipid accumulation, down regulated adipocyte hyperplasia related mRNA, and an enhanced mitochondrial fatty acid oxidation related gene expression pattern was confirmed. Furthermore, the analysis of condition media showed the absence of insulin resistance and immune cell-attracting inflammatory markers, such as LTB4, STAT-6, IL-4 and TNF-α levels. Macrophage treated with chia seed-treated adipocyte condition media effectively suppressed inflammatory cytokines (PAI-1, MCP-1, PGE-2 and TLR-4) linked with arterial vessels of cerebral, coronary and lower limb related immune disorders, such as atherosclerosis, stroke and thrombosis. This present observation majorly concludes that chia seed effectively controls fatty acid oxidation in adipocyte, which helps to inhibit obesity-linked inflammatory markers.

## Figures and Tables

**Table 1:** Primers sequence used in the Sybrgreen based real-time PCR.

Primer	Forward Sequence (5′ to 3′)	Reverse Sequence (5′ to 3′)
C/EBPα	CCGGGAGAACTCTAACTC	GATGTAGGCGCTGATGT
PPARγ	TCATAATGCCATCAGGTTTG	CTGGTCGATATCACTGGAG
HSL	CCTCATGGCTCAACTCC	GGTTCTTGACTATGGGTGA
LPL	AGGACCCCTGAAGACAG	GGCACCCAACTCTCATA
Adiponectin	CTACTGTTGCAAGCTCTCC	CTTCACATCTTTCATGTACACC
UCP-1	AGGCTTCCAGTACCATTAGGT	CTGAGTGAGGCAAAGCTGATTT
PPARγC_1_α	CCCTGCCATTGTTAAGACC	TGCTGCTGTTCCTGTTTTC
SREBP1c	GGAGCCATGGATTGCACATT	GCTTCCAGAGAGGAGGCCAG
PRDM16	CCCCACATTCCGCTGTGA	CTCGCAATCCTTGCACTCA
IL-1β	GCAAGGGCTTCAGGCAGGCCGCG	GGTCATTCTCCTGGAAGGTCTGTGGGC
IL-12β1	ATCAGGGACATCATCAAACCG	ACGCACCTTTCTGGTTACACTC
IL-6	TTCGGTCCAGTTGCCTTCTC	GAGGTGAGTGGCTGTCTGTG
IL-4	CAAACGTCCTCACAGCAACG	AGGCATCGAAAAGCCCGAAA
IL-33	TGAGACTCCGTTCTGGCCTC	CTCTTCATGCTTGGTACCCGAT
IKBKg	AACCAGCATCCAGATTGA C	GCCATCATCCGTTCTACC
TNFα	CTCCAGGCGGTGCCTTGTTC	CAGGCAGAAGAGCGTGGTG
NF-κB	GCGCTTCTCTGCCTTCCTTA	TCTTCAGGTTTGATGCCCCC
TLR-4	CCCTCATGACATCCCTATTTCA	CTCTCAGTACCAAGGTTGAGAGC
TGFBR2	TGCCGCCCTTCTTCCCCTC	GGAGCACAAGCTGCCCACTGA
Beta Actin	GATCTTGATCTTCATGGTGCTAGG	TTGTAACCAACTGGGACCATATGG

**Table 2:** Fatty acid composition analysis of chia seed extract using GC-MS showing 99%–95% similarity in the database.

Serial Number	Compound Name	Chia Seed Extract	
		RT *	Peak Area (%)
1	Hexadecenoic acid	24.457	9.49
2	9,12-octadecadienoic acid (ω-6 fatty acid)	27.623	21.35
3	9,12,15-octadecatrienoic acid (ω-3 fatty acid)	27.773	56.16
4	Methylstearate	28.222	3.94
5	Linoleic acid ethyl ester	28.799	7.56
6	Heptadecanoic acid	29.287	1.51

* RT = Retention Time; Peak area = the percentage of particular compound present in overall sample (100%); percentages have not rounded up to 100%, due to other constituents not listed.

**Table 3:** Effect of chia seed fatty acid extract on triglyceride, free glycerol, high density lipoprotein (HDL), low density lipoprotein (LDL) levels and lactate dehydrogenase (LDH) activity in matured adipocytes after 14 days.

Groups	Triglyceride (mg/dL)	Free Glycerol (mg/dL)	HDL (mg/dL)	LDL (mg/dL)	LDH Activity ¥
Vehicle control	6.2 ± 0.21	9.3 ± 0.25	0.42 ± 0.05	0.62 ± 0.03	0.21 ± 0.06
Chia seed 0.2 μg/mL	3.1 ± 0.26	5.2 ± 0.34 *	0.51 ± 0.06	0.53 ± 0.03	0.13 ± 0.03
Chia seed 0.4 μg/mL	2.3 ± 0.14 *	3.9 ± 0.29 *	0.85 ± 0.03 *	0.32 ± 0.02 *	0.09 ± 0.01 *
Orlistat, 6 µM	2.9 ± 0.13	5.4 ± 0.28 *	0.59 ± 0.02	0.39 ± 0.01 *	0.12 ± 0.03

Values are means ± SD (*n* = 6); ¥—one mU/mg protein of LDH activity angstrom (Å) one nmole of nicotinamide adenine dinucleotide (NADH) oxidized per minute per mg protein. *: *p* <0.05 compared with vehicle control.

## Supplementary Materials

The following are available online at https://www.mdpi.com/2304-8158/9/3/368/s1, S1. Showing the raw data for GC-MS results Agilent instrument software generated. S2. GC-MS instrumental methods and condition.
