# Supplementary material for: Beneficial Fatty Acid Ratio of Salvia hispanica L. (Chia Seed) Potentially Inhibits Adipocyte Hypertrophy, and Decreases Adipokines Expression and Inflammation in Macrophage
Source: Foods. 2020 Mar 22;9(3):368. doi: 10.3390/foods9030368 (PMC7143507; doi:10.3390/foods9030368)
Supplement: Supplementary file 1 [file foods-09-00368-s001.zip › Chia seed- fatty acid GCMS.pdf]

```
File        :C:\msdchem\1\data\2016\July-16\30-07-16\E-C-260-07P.D
Operator    :
Acquired    : 30 Jul 2016   18:17      using AcqMethod FATTYACIDCOMPOSITION_PROCESSING.M
Instrument :    GCMS
Sample Name: Chia
Misc Info   :
Vial Number: 2
```

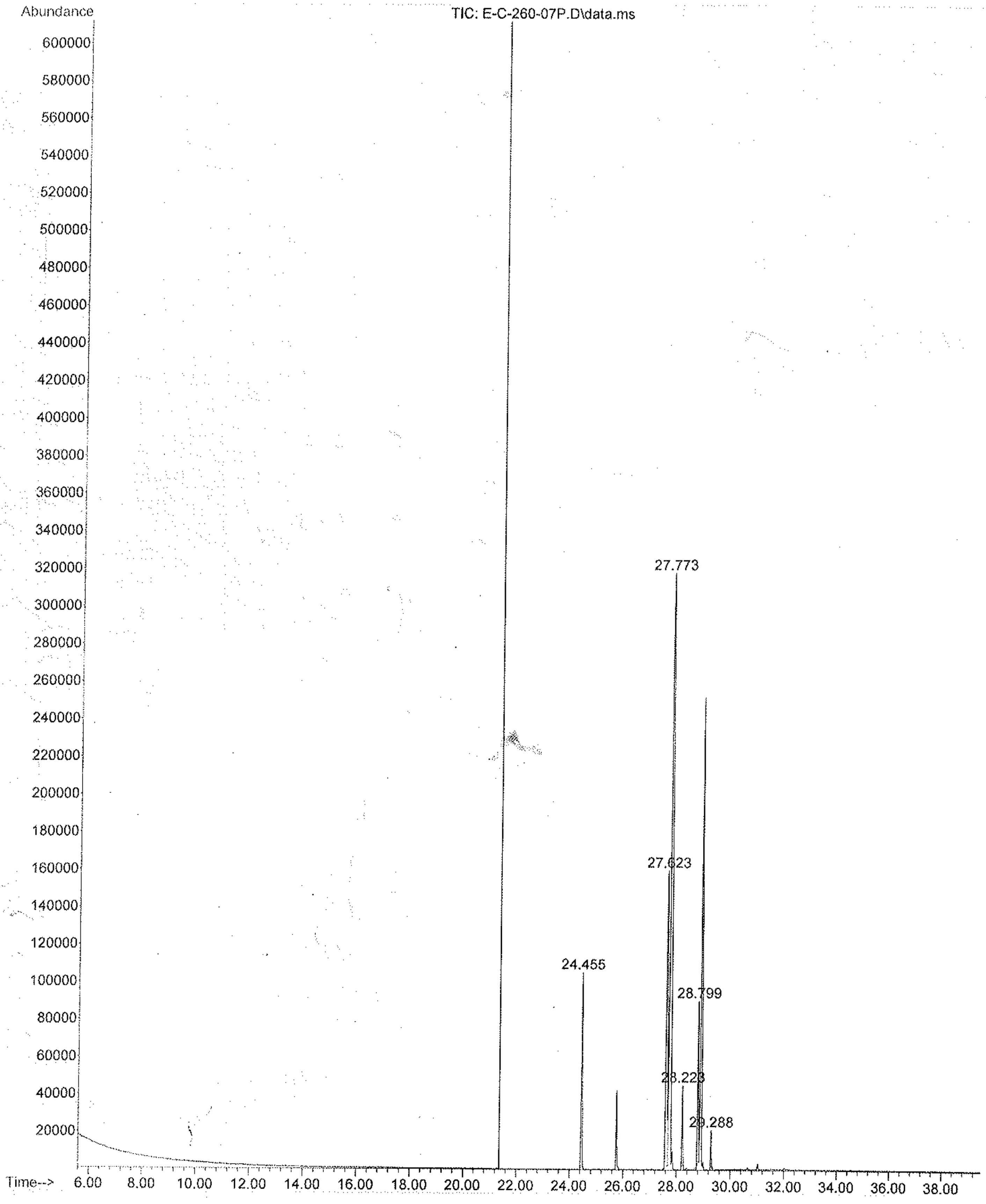

Library Search Report

Data Path : C:\msdchem\1\data\2016\July-16\30-07-16\
Data File : E-C-260-07P.D
Acq On : 30 Jul 2016 18:17
Operator :
Sample : Chia
Misc :
ALS Vial : 2 Sample Multiplier: 1

Search Libraries: C:\Database\NIST11.L Minimum Quality: 0

Unknown Spectrum: Apex
Integration Events: ChemStation Integrator - E-C-260-07P.E

Pk#	RT	Area%	Library/ID	Ref#	CAS#	Qual
1	24.457	9.49	C:\Database\NIST11.L			
			Hexadecanoic acid, methyl ester	119400	000112-39-0	97
			Hexadecanoic acid, methyl ester	119405	000112-39-0	97
			Pentadecanoic acid, 14-methyl-, methyl ester	119423	005129-60-2	97
2	27.623	21.35	C:\Database\NIST11.L			
			9,12-Octadecadienoic acid, methyl ester	139708	002462-85-3	99
			9,12-Octadecadienoic acid (Z,Z)-, methyl ester	139726	000112-63-0	99
			Methyl 10-trans,12-cis-octadecadienoate	139709	1000336-44-2	99
3	27.773	56.16	C:\Database\NIST11.L			
			9,12,15-Octadecatrienoic acid, methyl ester, (Z,Z,Z)-	138095	000301-00-8	99
			7,10,13-Hexadecatrienoic acid, methyl ester	114197	056554-30-4	95
			7,10,13-Hexadecatrienoic acid, methyl ester	114196	056554-30-4	91
4	28.222	3.94	C:\Database\NIST11.L			
			Methyl stearate	143131	000112-61-8	98
			Methyl stearate	143126	000112-61-8	98
			Methyl stearate	143129	000112-61-8	98
5	28.800	7.56	C:\Database\NIST11.L			
			Linoleic acid ethyl ester	151450	000544-35-4	99
			9,12-Octadecadienoic acid, ethyl ester	151471	007619-08-1	99
			9,12-Octadecadienoic acid (Z,Z)-	127648	000060-33-3	91
6	29.287	1.51	C:\Database\NIST11.L			
			Heptadecanoic acid, ethyl ester	143161	014010-23-2	87
			Heptadecanoic acid, ethyl ester	143163	014010-23-2	83
			Octadecanoic acid, ethyl ester	154935	000111-61-5	83

Library Search Report

Data Path : C:\msdchem\1\data\2016\July-16\30-07-16\
Data File : E-C-260-07P.D
Acq On : 30 Jul 2016 18:17
Operator :
Sample : Chia
Misc :
ALS Vial : 2 Sample Multiplier: 1

Search Libraries: C:\Database\NIST11.L Minimum Quality: 0

Unknown Spectrum: Apex
Integration Events: ChemStation Integrator - E-C-260-07P.E

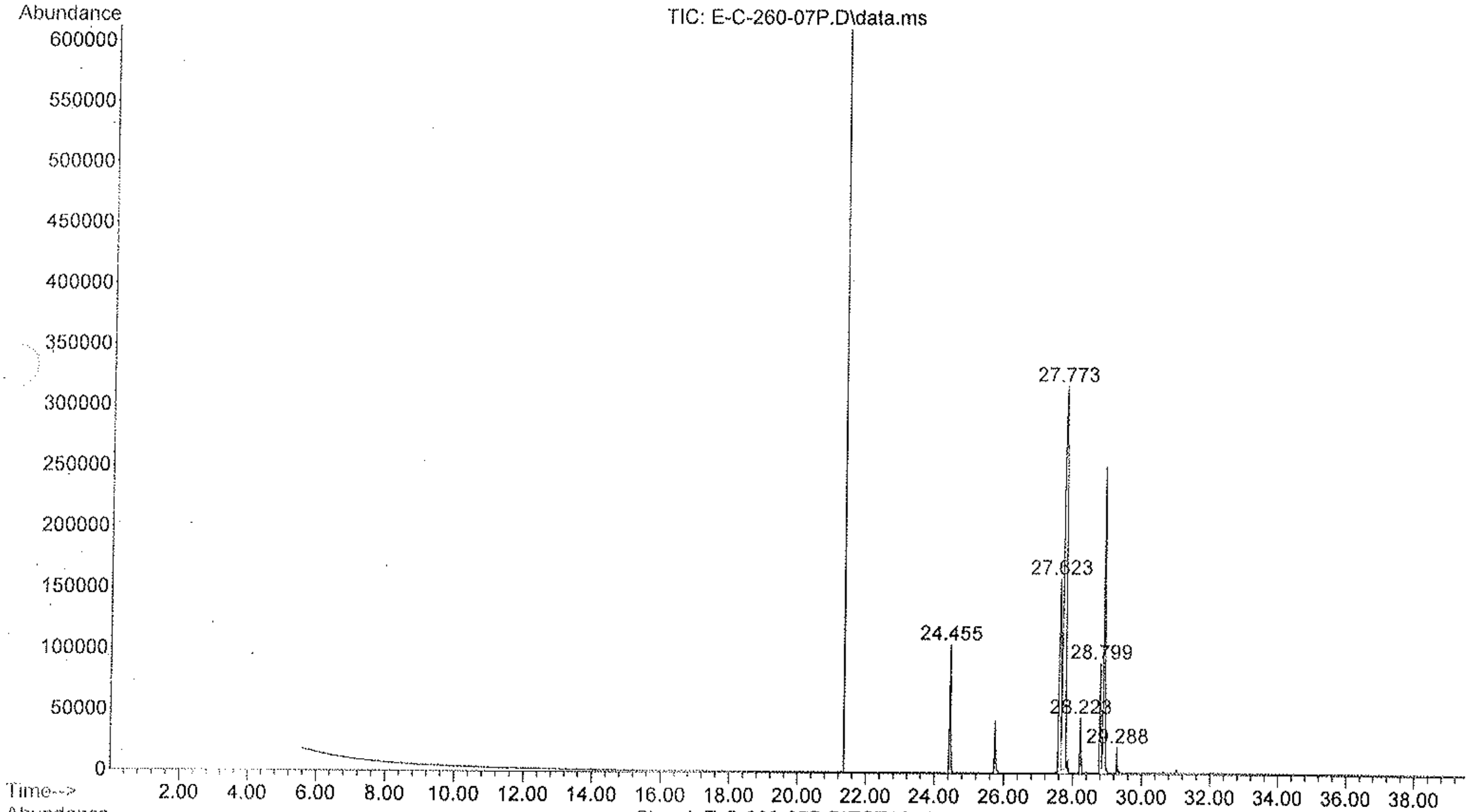

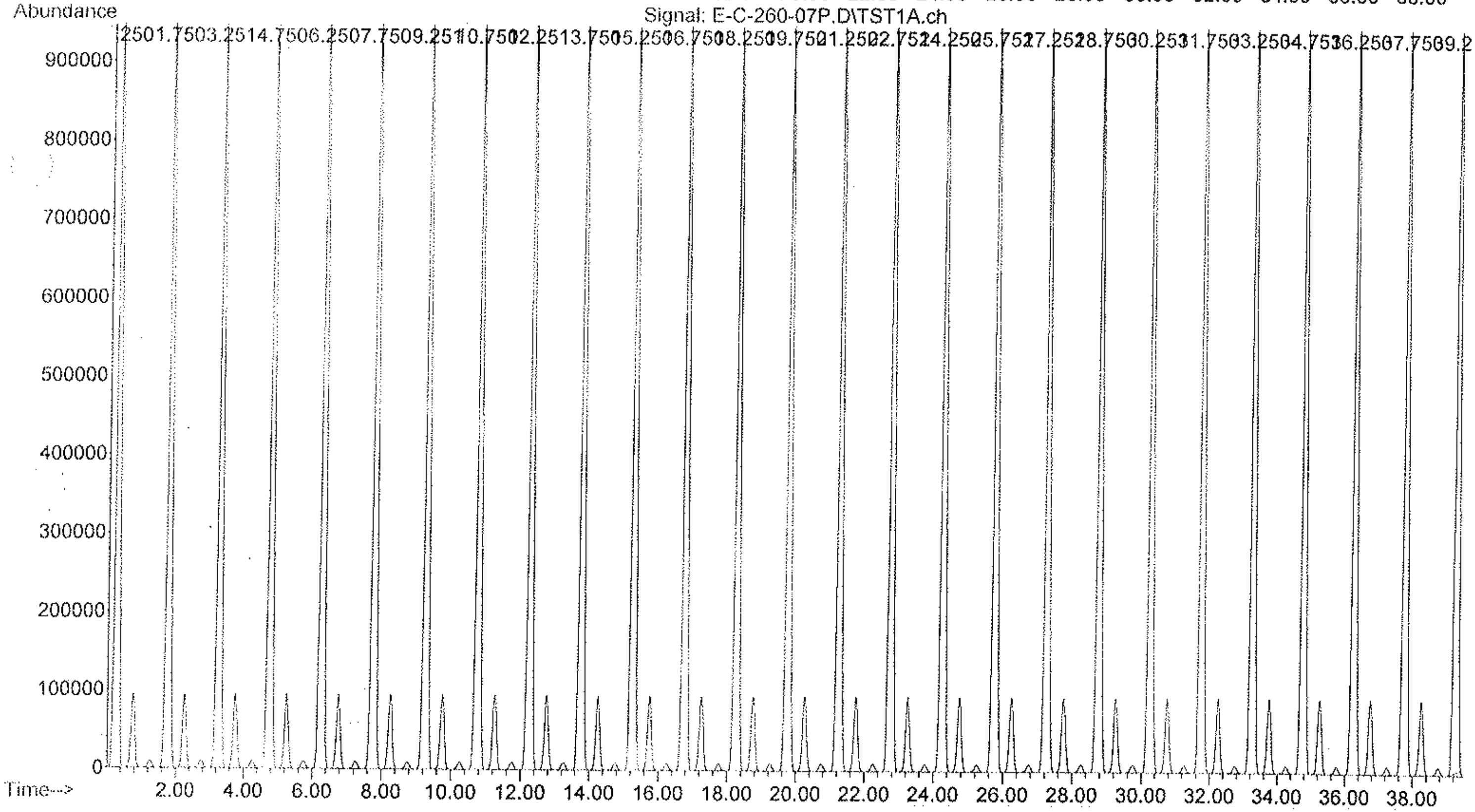

Unknown Spectrum based on Apex

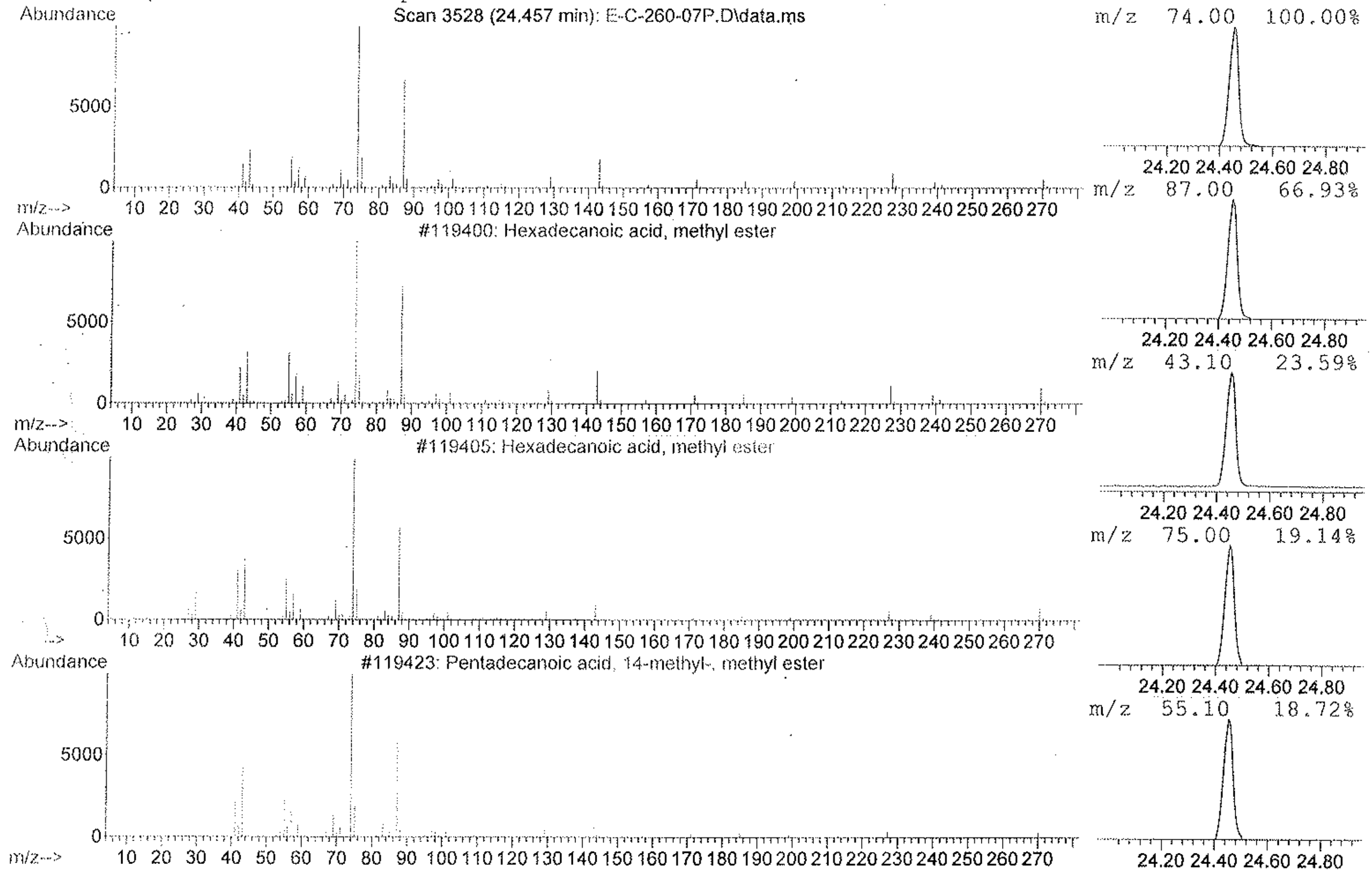

Data File: C:\msdchem\1\data\2016\July-16\30-07-16\E-C-260-07P.D
Sample : Chia

Peak Number: 1 at 24.457 min Area: 2556692 Area % 9.49

The 3 best hits from each library.	Ref\#	CAS\#	Qual
C:\Database\NIST11.L			
1 Hexadecanoic acid, methyl ester	119400	000112-39-0	97
2 Hexadecanoic acid, methyl ester	119405	000112-39-0	97
3 Pentadecanoic acid, 14-methyl-, ...	119423	005129-60-2	97

Library Search Report - ChemStation Integrator

Unknown Spectrum based on Apex

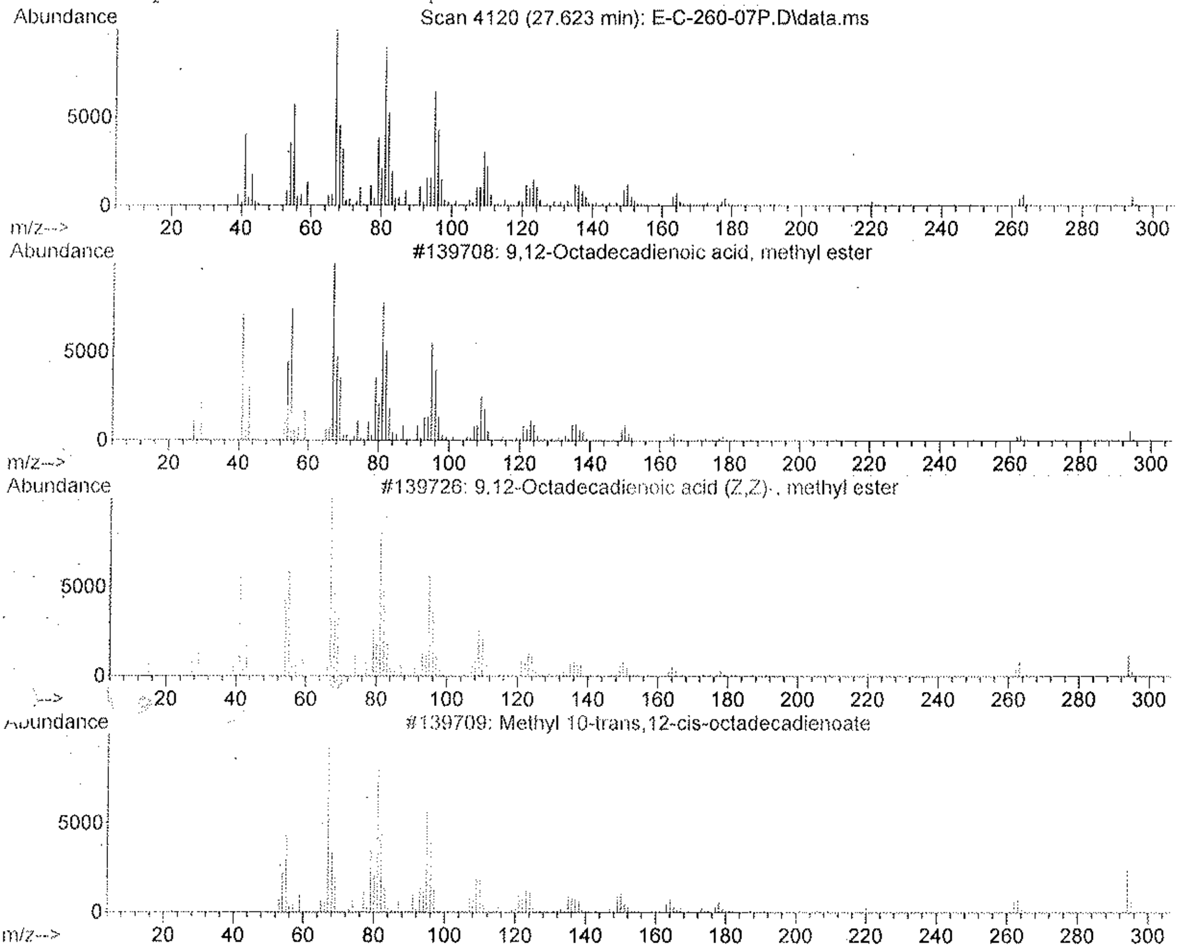

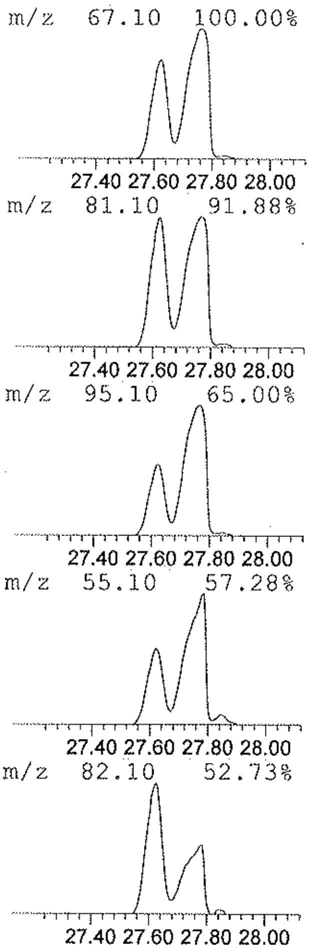

Data File: C:\msdchem\1\data\2016\July-16\30-07-16\E-C-260-07P.D
Sample : Chia

Peak Number: 2 at 27.623 min Area: 5752494 Area % 21.35

The 3 best hits from each library.	Ref\#	CAS\#	Qual
C:\Database\NIST11.L			
1 9,12-Octadecadienoic acid, methy...	139708	002462-85-3	99
2 9,12-Octadecadienoic acid (Z,Z)-...	139726	000112-63-0	99
3 Methyl 10-trans,12-cis-octadecad...	139709	1000336-44-2	99

Unknown Spectrum based on Apex

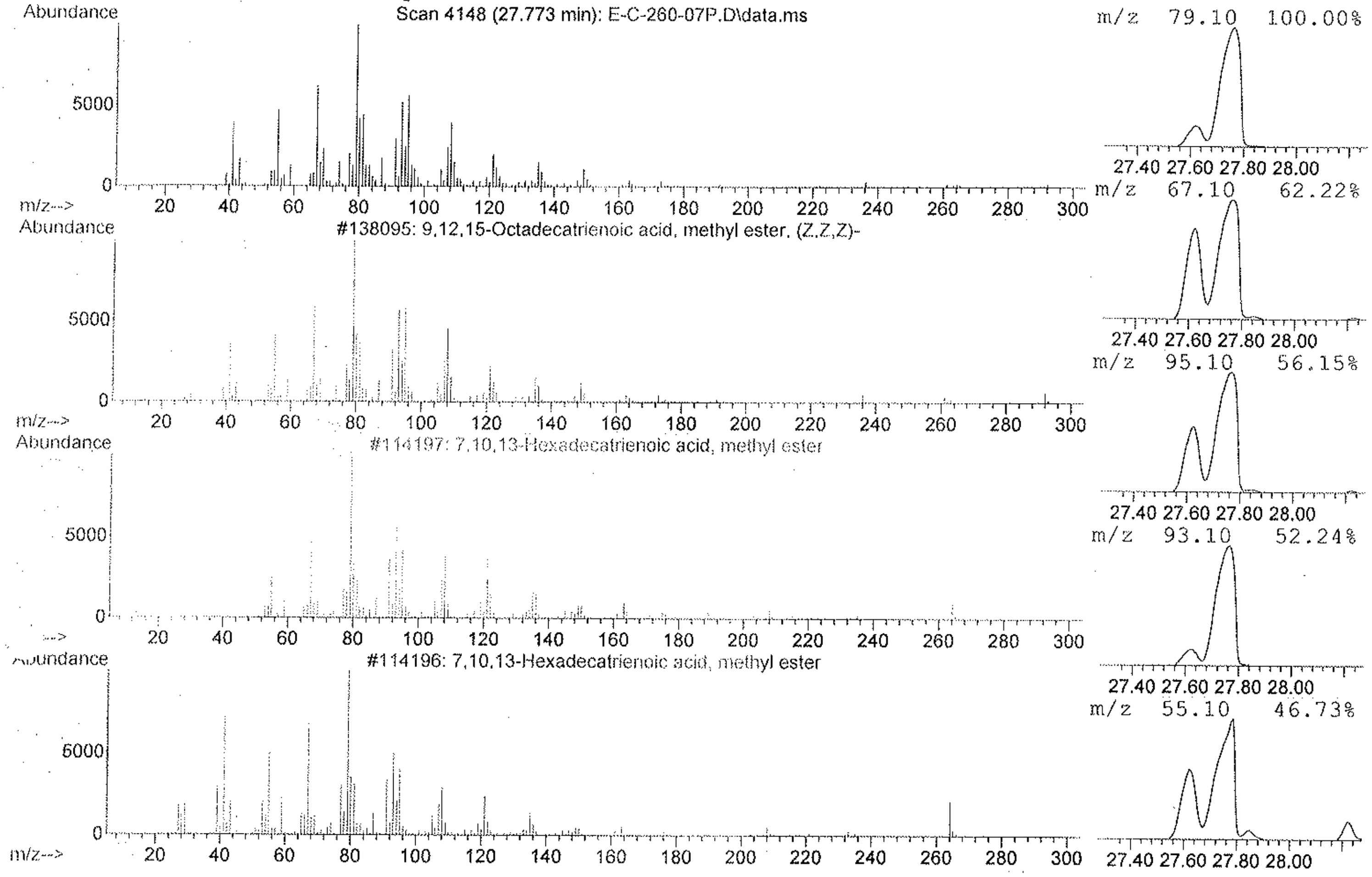

Data File: C:\msdchem\1\data\2016\July-16\30-07-16\E-C-260-07P.D
Sample : Chia

Peak Number: 3 at 27.773 min Area: 15132970 Area % 56.16

The 3 best hits from each library.	Ref\#	CAS\#	Qual
C:\Database\NIST11.L			
1 9,12,15-Octadecatrienoic acid, m...	138095	000301-00-8	99
2 7,10,13-Hexadecatrienoic acid, m...	114197	056554-30-4	95
3 7,10,13-Hexadecatrienoic acid, m...	114196	056554-30-4	91

Library Search Report - ChemStation Integrator

Unknown Spectrum based on Apex

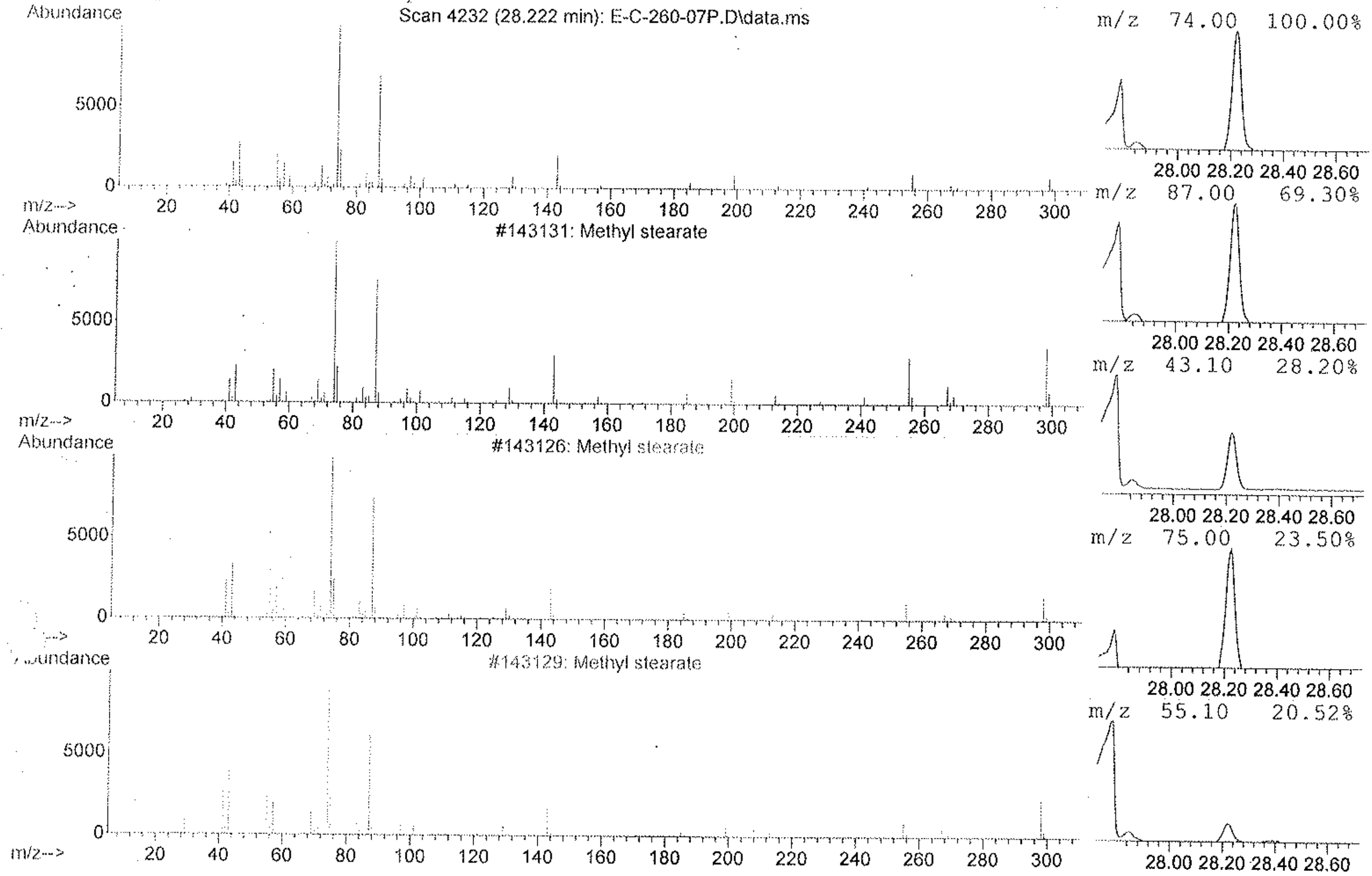

Data File: C:\msdchem\1\data\2016\July-16\30-07-16\E-C-260-07P.D
Sample : Chia

Peak Number: 4 at 28.222 min Area: 1060675 Area % 3.94

The 3 best hits from each library.	Ref\#	CAS\#	Qual
C:\Database\NIST11.L			
1 Methyl stearate	143131	000112-61-8	98
2 Methyl stearate	143126	000112-61-8	98
3 Methyl stearate	143129	000112-61-8	98

Unknown Spectrum based on Apex

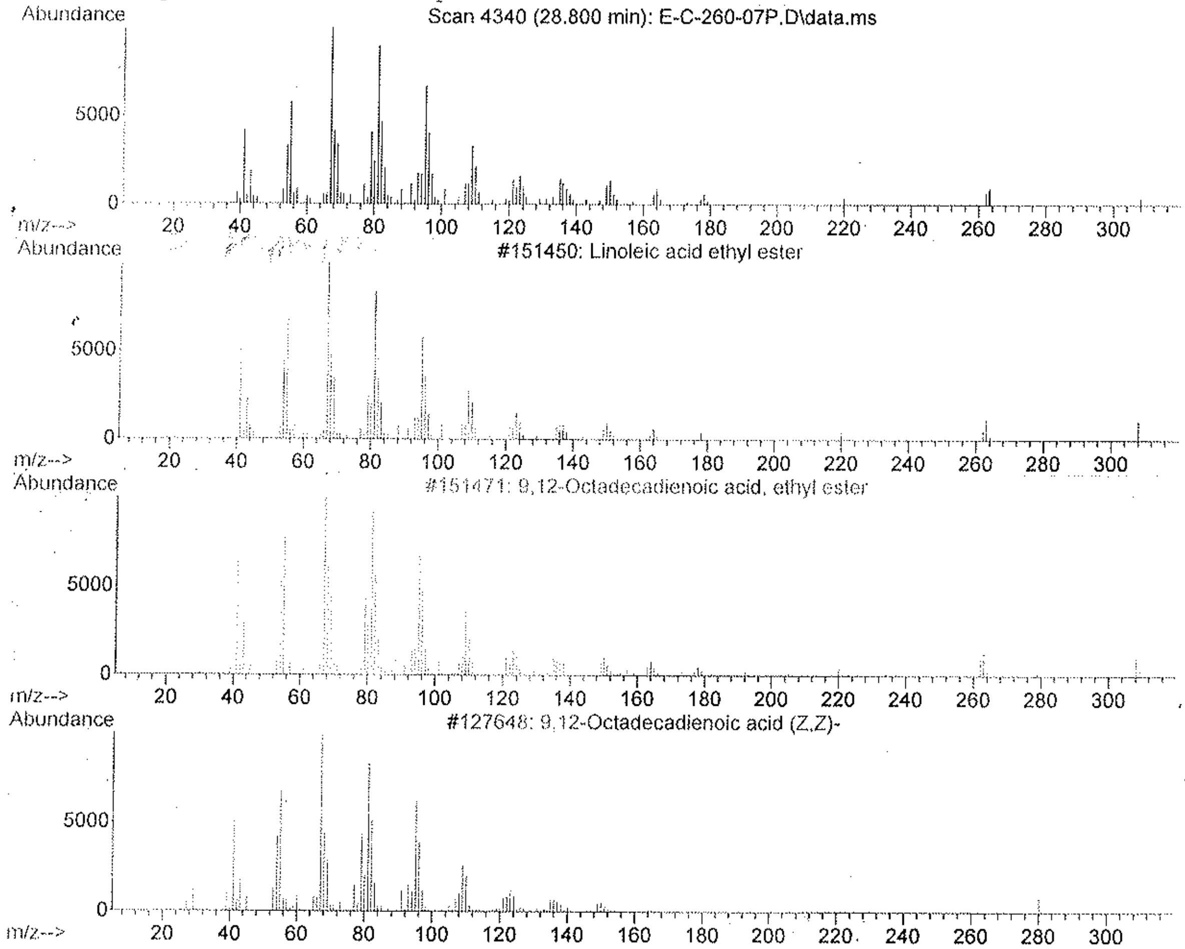

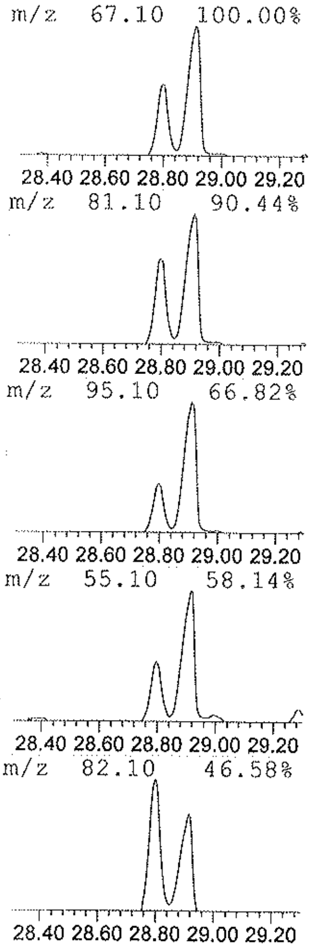

Data File: C:\msdchem\1\data\2016\July-16\30-07-16\E-C-260-07P.D
Sample : Chia

Peak Number: 5 at 28.800 min Area: 2036278 Area % 7.56

The 3 best hits from each library.	Ref\#	CAS\#	Qual
C:\Database\NIST11.L			
1 Linoleic acid ethyl ester	151450	000544-35-4	99
2 9,12-Octadecadienoic acid, ethyl...	151471	007619-08-1	99
3 9,12-Octadecadienoic acid (Z,Z)-	127648	000060-33-3	91

Unknown Spectrum based on Apex

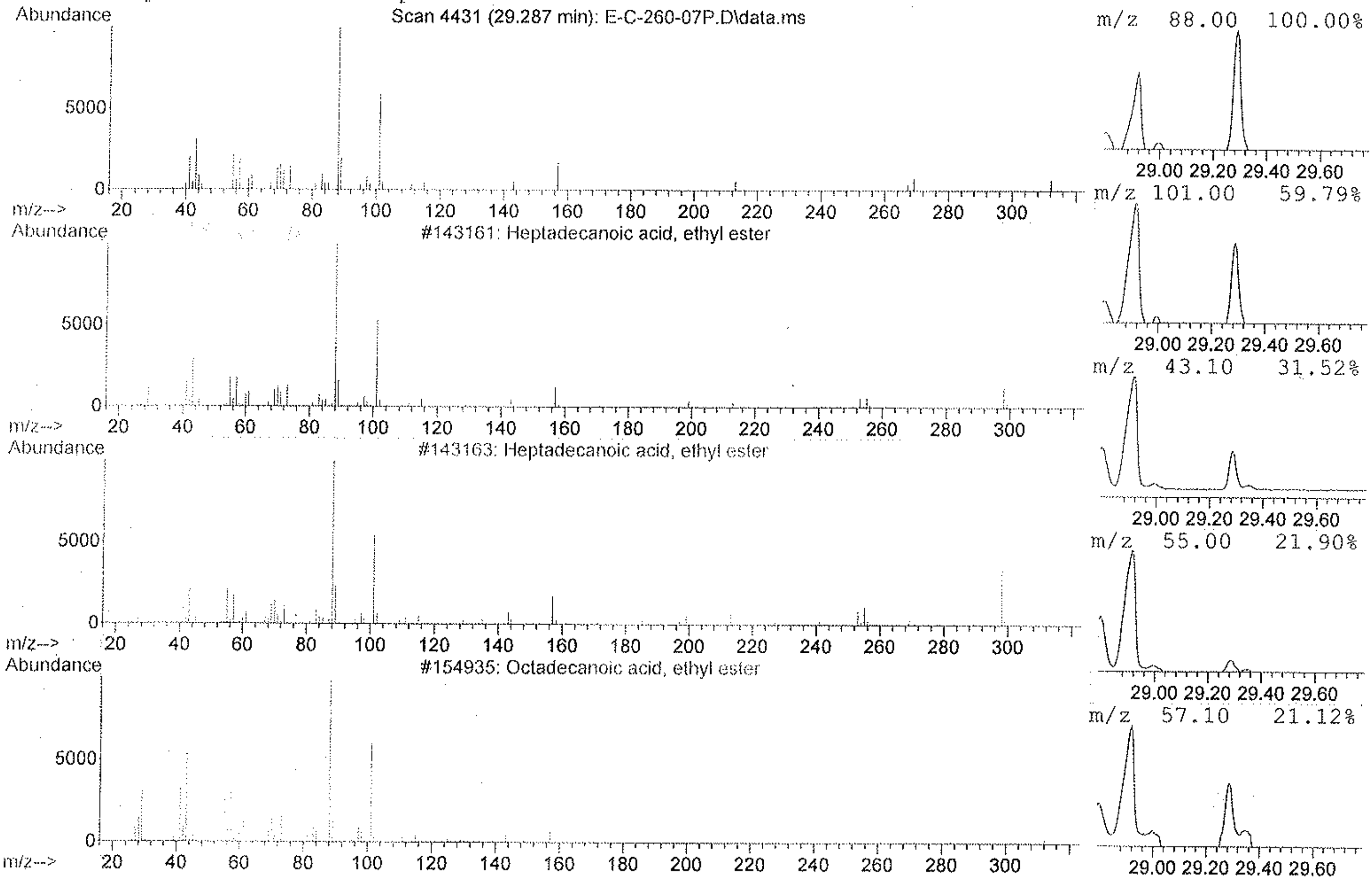

Data File: C:\msdchem\1\data\2016\July-16\30-07-16\E-C-260-07P.D
Sample : Chia

Peak Number: 6 at 29.287 min Area: 405825 Area % 1.51

The 3 best hits from each library.	Ref\#	CAS\#	Qual
C:\Database\NIST11.L			
1 Heptadecanoic acid, ethyl ester	143161	014010-23-2	87
2 Heptadecanoic acid, ethyl ester	143163	014010-23-2	83
3 Octadecanoic acid, ethyl ester	154935	000111-61-5	83
