# Supplementary material for: Beneficial Fatty Acid Ratio of Salvia hispanica L. (Chia Seed) Potentially Inhibits Adipocyte Hypertrophy, and Decreases Adipokines Expression and Inflammation in Macrophage
Source: Foods. 2020 Mar 22;9(3):368. doi: 10.3390/foods9030368 (PMC7143507; doi:10.3390/foods9030368)
Supplement: Supplementary file 1 [file foods-09-00368-s001.zip › GC MS instrumental methods and condition-Agilent.doc]

**GC-MS Method Condition:**

Instrument Name: GC MS

Instrument Make: Agilent

Instrument Model: 7890 A, MS 5975

Column used: J&W 5 Ms: 30m x 0.25 mm ID x 1.4 Film Thickness

Column Flow: 1.0 ml per min @ constant Flow.

Injection Volume: 3 ul

Injection Temperature: 250^0^C, Splitless

Oven programme :

Rate/deg	temp ^0^C	Hold (min)

Step 1	50	3.0

Step 2	10	100	3.0

Step 3	10	300	4.0 (35 min)

Aux temp: 250^0^C

Carrier gas: Helium

**MS parameters**: Scan Mode

Solvent Delay: 2.0 minutes

Mass Range: 35 to 800

Ion source: EI (Electron Impact)

Source MS Temp: 230^0^C

MS Quad Temp: 150^0^C

Fixed Electron Energy: -70eV

**Library Used**: NIST 11
